# Correction: Banjac et al. The Effects of Different Doses of Sildenafil on Coronary Blood Flow and Oxidative Stress in Isolated Rat Hearts. *Pharmaceuticals* 2023, *16*, 118

**DOI:** 10.3390/ph17040439

**Published:** 2024-03-29

**Authors:** Nada Banjac, Velibor Vasović, Nebojša Stilinović, Ana Tomas, Lucija Vasović, Nikola Martić, Dušan Prodanović, Vladimir Jakovljević

**Affiliations:** 1 Medical Faculty, University of Banja Luka, 78000 Banja Luka, Republika Srpska, Bosnia and Herzegovina; 2 Department of Pharmacology, Toxicology and Clinical Pharmacology, Faculty of Medicine, University of Novi Sad, 21000 Novi Sad, Serbia; 3 Faculty of Medicine, University of Novi Sad, 21000 Novi Sad, Serbia; 4 Department of Physiology, Faculty of Medical Sciences, University of Kragujevac, 34000 Kragujevac, Serbia

## Change of the Corresponding Author

Prof. Velibor Vasović was not included as the corresponding author in the original publication [1]. The authors state that the scientific conclusions are unaffected. This correction was approved by the Academic Editor. The original publication has also been updated.
